# Association of ghrelin and leptin with reproductive hormones in constitutional delay of growth and puberty

**DOI:** 10.1186/1477-7827-8-153

**Published:** 2010-12-22

**Authors:** Mervat M El-Eshmawy, Ibrahim A Abdel Aal, Amany K El hawary

**Affiliations:** 1 Internal Medicine Department, Mansoura Specialized Medical Hospital, Faculty of Medicine, Mansoura University, Egypt; 2 Clinical Pathology Department, Faculty of Medicine, Mansoura University, Mansoura, Egypt; 3 Pediatric Department, Mansoura pediatric Hospital, Faculty of Medicine, Mansoura University, Mansoura, Egypt

## Abstract

**Background:**

Constitutional delay of growth and puberty (CDGP) is a variation of the onset and timing of pubertal development without a defined endocrine abnormality. Recently published studies indicate that leptin and ghrelin play a role in puberty initiation and progress. They have been implicated in regulation of GnRH secretion, with ghrelin having inhibitory and leptin, facilitatory effects. We hypothesized that elevated ghrelin and reduced leptin concentrations could be implicated in altering the tempo of puberty in adolescents with CDGP. So in the current study we evaluate variations in leptin and ghrelin levels in adolescent boys with CDGP, the relationships between both hormones and reproductive hormones including LH, FSH and testosterone were also evaluated.

**Methods:**

The study enrolled 23 adolescent boys with CDGP and 20 healthy controls matched for age and sex. Weight, height, BMI, testicular volume, bone age, bone age delay, serum FSH, LH, testosterone, leptin and ghrelin were assessed.

**Results:**

Adolescent boys with CDGP had significantly lower leptin and higher ghrelin than normal controls. Leptin was positively correlated with BMI, bone age, testicular volume, FSH, LH and testosterone and negatively correlated with delayed bone age and ghrelin. Ghrelin was negatively correlated with BMI, bone age, testicular volume, FSH, LH and testosterone. With multiple regression analysis BMI, FSH, LH, testosterone and ghrelin remained independently correlated with leptin while BMI, LH and testosterone remained independently correlated with ghrelin.

**Conclusion:**

Elevated serum ghrelin and decreased leptin concentrations and their associations with reproductive hormones may explain the sexual immaturity in adolescent boys with CDGP.

## Background

Constitutional delay of growth and puberty (CDGP) is a disorder occurring in healthy adolescents who have short stature compared with their peers, delay in bone maturation and delayed puberty [1]. Most children with CDGP begin to deviate from the normal growth curve before age 2 yr, subsequently grow at a relatively normal velocity, and then have a delayed pubertal growth spurt [2]. In boys with CDGP, a testicular volume of 3-4 ml is first reached when they are more than 13.7 years old. The sleep related Luteinizing hormone (LH) increase that characterizes the onset of puberty, is normally present in CDGP. The LH response to Luteinizing hormone releasing hormone (LHRH) analogues is intermediate between that of hypogonadal patients and normal pubertal children [3]. CDGP represents the extreme tail of the normal distribution, aggregates in families [4] and is much more common in boys [5]. A suspected diagnosis of CDGP can be definitely confirmed only when puberty and the pubertal growth spurt finally do occur spontaneously, more than two standard deviations later than the normal mean age.

Pubertal development and fertility are determined by a multi-hormonal effect. Puberty is characterized by increasing concentrations of gonadal estradiol in girls and testosterone in boys, driven by increasing concentrations of pituitary gonadotrophins which are, in turn, regulated by gonadotrophin-releasing hormone (GnRH) released by hypothalamic neurons [6,7]. A functional defect in any of the components of this hormonal complex directly affects puberty and reproduction in either gender. Recent research added two new members to this hormonal complex, namely leptin and ghrelin [8,9], which are secreted by adipose tissue and gastrointestinal tract, respectively. Besides their effect on carbohydrate and fat metabolism and appetite, these hormones acting on the hypothalamic-pituitary-gonadal axis, exert various effects on reproductive function [7].

Leptin, an adipocyte-derived hormone, is a key regulator of energy homeostasis and adiposity. It acts directly on hypothalamic nuclei to suppress food intake and increase energy expenditure [10]. In addition, leptin has been proposed to contribute to hypothalamic-pituitary-gonadal function [11]. Indeed, leptin is clearly significant in pubertal development and progression in humans [12]; congenital leptin deficiency due to mutations in either the leptin gene or the leptin receptor gene, is associated with early-onset obesity and no pubertal development [12,13].

Ghrelin is a 28-amino acid peptide produced in a variety of human tissues; however the major source of circulating ghrelin is the stomach [14]. It regulates a large array of endocrine and non endocrine functions, including the control of growth hormone (GH) secretion, food intake, energy balance and control of adiposity [15]. Ghrelin is the endogenous ligand for the GH secretagogue receptor (GHS-R) [14]. Together, ghrelin and growth hormone releasing hormone (GHRH) synergistically increase GH levels [16]. Ghrelin stimulates appetite and induces a positive energy balance that can lead to weight gain [17]. In addition, ghrelin reduces GnRH secretion in the pre-pubertal period [18].

The aim of the present study was to investigate the variations in leptin and ghrelin levels in adolescent boys with CDGP, the relationships between both hormones and reproductive hormones including LH, Follicle stimulating hormone (FSH) and testosterone were also evaluated.

## Methods

The study comprised twenty three adolescent boys aged 14-16 years with CDGP (Table 1).

**Table 1:** Subjects characteristics

Characteristics	Adolescents with CDGP (n = 23)	Normal control (n = 20)	P-value
**Age (years)**	15.1 + 0.67	15.1 + 0.72	0.9
**Weight (kg)**	38.5 + 3.1	64.3 + 13.36	< 0.001*
**Standing height (cm)**	147.8 + 5.9	169.55 + 9.9	< 0.001*
**BMI (kg/m^2^)**	17.6 + 0.9	23.4 + 2.2	< 0.001*
**Testicular volume (ml)**	2.6 + 0.5	11.6 + 2	< 0.001*
**Bone age (years)**	12.7 + 0.5	15.1 + 0.67	< 0.001*
**Bone age delay (years)**	2.3 + 0.4	-	
**FSH (iu/ml)**	0.4 + 0.08	5.1 + 0. 7	< 0.001*
**LH (iu/ml)**	0.2 + 0.06	4.3 + 0.7	< 0.001*
**Testosterone (ng/ml)**	0.06 + 0.02	4.5 + 3.2	< 0.001*
**Leptin (ng/ml)**	3.06 + 0.6	7.03 + 2.5	< 0.001*
**Ghrelin (ng/ml)**	276.4 + 58.9	106.5 + 29.6	< 0.001*

CDGP: Constitutional delay of growth and puberty; BMI: Body mass index; FSH: Follicle stimulating hormone; LH: Luteinizing hormone; Data are expressed as mean ± standard deviation; *P is significant if < 0.05

The diagnosis of CDGP was based on the following criteria:

1) Short stature (height less than 2SD below the mean)

2) Delayed puberty (onset delayed by more than 2SD- that is, has not achieved 4 ml testes until aged 14 years).

3) Bone age below the 10^th ^centile for chronological age (delayed by more than 1.5 years).

3) Absence of other causes of delayed puberty on history, examination and investigations. None of the participants was taking any medication.

Height and weight were measured using standard procedures. Body mass index (BMI) was calculated from the weight in kilograms divided by the square of the height in meters. Pubertal stage was assessed according to the method of Tanner and Whitehouse [19]. Skeletal age was determined on left wrist radiographs using Greulich and Pyle method. Bone age delay was noted as the difference between skeletal and chronological age. Twenty healthy adolescent boys matched for age in the mid and late pubertal stage (3-5 Tanner stage) were evaluated as controls.

All adolescents with CDGP subsequently attain puberty either spontaneously (15 boys) or after induction of puberty (8 boys). They progress through puberty with increasing testicular volumes and pubertal staging.

### Hormone assays

To assess episodic hormone secretion, 3 ml fasting blood samples were collected at 20 minutes intervals for 3 hours and the separated sera were mixed for the quantitative determination of ghrelin, leptin, FSH, LH and testosterone. Seum leptin levels were estimated by an enzyme immunoassay test that follows a typical two-Step capture (sandwitch) assay. (Diagnostic Biochem Canada Inc, DBC). Serum ghrelin levels were estimated by enzyme immunoassay kit designed to detect a specific peptide based on the principle of competitive enzyme immunoassay (DRG International inc., USA). FSH, LH and testosterone were determined using ECLIA electro-chemiluminescent immunoassay through automated auto analyzer Elec Sys 2010 (Roche, Germany).

All subjects signed an informed consent to be included in our study. The study was approved by the local ethical committee.

### Statistical analysis

Data were analyzed using SPSS statistical package version 10 (SPSS, Inc., Chicago, IL, USA). The quantitative data were presented as a mean and a standard deviation. For the qualitative data, student t-test was used to compare between two groups. Simple regression analysis was performed with leptin and ghrelin as the dependent variables and all other parameters as independent variables. Multiple regression analysis was also performed with leptin and ghrelin as the dependent variables and BMI, testicular volume, bone age, bone age delay, FSH, LH and testosterone as independent variables.

P value of < 0.05 indicates significant results.

## Results

### Clinical and biochemical characteristics of the study subjects

Baseline characteristics of the adolescents with CDGP and age and sex matched controls are given in Table 1. Adolescent boys with CDGP had significantly lower body weight, height, BMI, testicular volume and bone age than controls. Bone age delay of the adolescent boys with CDGP was 2.3 + 0.4 years.

FSH, LH, testosterone and leptin levels were significantly lower (p < 0.001) in adolescent boys with CDGP than controls. Ghrelin levels were significantly higher in the adolescent boys with CDGP than controls (p < 0.001).

### Correlations between leptin levels and all other parameters

Leptin levels were positively correlated with height, weight, testicular volume, bone age, FSH (p = 0.04), LH (p = 0.038) and testosterone (p = 0.039), and negatively correlated with bone age delay (p = 0.012) and ghrelin (p = 0.005) Table 2.

**Table 2:** Correlation between leptin and cinical and biochemical parameters in adolescents with CDGP

Parameters	R	P*- *value
**Age (years)**	0.13	0.5
**Weight (kg)**	0.62	< 0.001*
**Height (cm)**	0.79	0.002*
**BMI (kg/m^2^)**	0.87	< 0.001*
**Testicular volume(ml)**	0.37	0.049*
**Bone age(years)**	0.52	0.011*
**Bone age delay(years)**	-0.47	0.012*
**FSH (iu/ml)**	0.44	0.04*
**LH (iu/ml)**	0.42	0.038*
**Testosterone (ng/ml)**	0.43	0.039*
**Ghrelin (ng/ml)**	- 0.56	0.005*

CDGP: Constitutional delay of growth and puberty; BMI: Body mass index;

FSH: Follicle stimulating hormone; LH: Luteinizing hormone; *P is significant if < 0.05

### Correlations between ghrelin levels and all other parameters

Ghrelin levels were negatively correlated with height, weight, BMI, testicular volume, bone age, FSH (p = 0.03), LH (p = 0.01) and testosterone (p = 0.032), and positively correlated with bone age delay (p = 0.008) Table 3.

**Table 3:** Correlation between ghrelin and cinical and biochemical parameters in adolescents with CDGP

Parameters	R	P*- *value
**Age (years)**	- 0.15	0.4
**Weight (kg)**	- 0.39	0.03*
**Height (cm)**	- 0.48	0.02*
**BMI (kg/m^2^)**	- 0.46	0.02*
**Testicular volume(ml)**	- 0.43	0.04*
**Bone age(years)**	- 0.35	0.043*
**Bone age delay(years)**	0.53	0.008*
**FSH (iu/ml)**	- 0.45	0.03*
**LH (iu/ml)**	- 0.49	0.01*
**Testosterone (ng/ml)**	- 0.39	0.032*

CDGP: Constitutional delay of growth and puberty; BMI: Body mass index;

FSH: Follicle stimulating hormone; LH: Luteinizing hormone; *P is significant if < 0.05

### Multiple regression analysis with leptin and ghrelin as the dependent variables and other parameters as the independent variables

A multiple regression analysis to assess the independent effect of studied variables (BMI, testicular volume, bone age, bone age delay, FSH, LH, testosterone) on both leptin and ghrelin levels was also performed. BMI (β = 0.77, p = 0.002), FSH ((β = 0.64, p = 0.03), LH ((β = 0.66, p = 0.02), testosterone (β = 0.41, p = 0.009) and ghrelin (β = - 0.55, p = 0.01) remained independently correlated with leptin levels while BMI (β = -1.13, p = 0.005), LH (β = - 0.62, p = 0.01) and testosterone (β = - 0.82, p = 0.007), remained independently correlated with ghrelin levels.

## Discussion

Puberty constitutes a distinct developmental stage characterized by physiological, anatomical and psychological alterations and comprises a preparatory step for reproduction. Age at puberty is determined by genetic and environmental factors. CDGP is a variation of the onset and timing of pubertal development without a defined endocrine abnormality. Recently published results showed that both leptin and ghrelin have a role in GnRH production at different reproductive stages [18,20,21]; they showed opposing effects on pulsatile GnRH secretion [18]. We hypothesized that low leptin and high ghrelin concentrations could be implicated in altering the tempo of puberty in adolescents with CDGP.

In the present study, leptin levels were significantly lower in adolescent boys with CDGP than in normal controls. Our results also showed significant positive correlations between leptin levels and weight, BMI, FSH, LH and testosterone. These results are consistent with previous reports of low leptin concentrations in boys with CDGP [22,23]. Bideci et al. [22] also demonstrated a statistically significant relationship between leptin levels and hight, weight, BMI and bone age, they suggested that the reason of short stature and pubertal delay may be the decrease in weight which also the cause of low leptin level.

Leptin is clearly significant in pubertal development and progression [12,24]. Mantzoros et al. [25] showed that leptin levels increase by approximately 50% before the onset of puberty in healthy boys before testosterone, LH, or FSH increases and decrease to baseline values after the initiation of puberty, which may indicate a role for leptin in pubertal initiation. However, Ozata et al. [26] found that leptin seems not to be a primary signal for the onset of puberty: instead it may act in a permissive way as one of several metabolic factors. In parallel with the ob/ob mouse, humans who have rare mutations of the leptin gene and are very obese remain prepubertal [12] unless they are given recombinant leptin which restores pulsatile gonadotropin secretion [27]. Furthermore, obese children have early puberty that could be the effect of high circulating levels of leptin [28].

Leptin regulates the hypothalamus-pituitary-gonad axis at both the central and gonadal levels [29]. Leptin stimulates LH and FSH in pituitary gonadotrophes [30] and also implicated in steroidogenesis in the gonads [31]. Stimulation of reproductive neuroendocrine output is also associated with increased circulating levels of leptin [32]. Adipocytes specifically bind androgens [33] and appear to carry androgen receptors [34]. Morelli et al. [35] suggested that androgens (adequate hormonal status) could have a positive effect on GnRH neuronal activity by synergizing with leptin (adequate energy status) in the regulatory mechanisms required for reproductive and sexual fitness.

As expected we found significant elevation of ghrelin concentrations in adolescent boys with CDGP compared to healthy controls. Our results also showed significant negative correlations between ghrelin and BMI, LH and testosterone. Our results were in parallel with many studies which demonstrated negative and significant correlations between ghrelin and LH and testosterone [15,36,37]. Also, Pomerants et al. [38] found decreased fasting ghrelin concentration and negative correlations between its level and body weight, BMI, lean body mass and testosterone concentrations in healthy pubertal boys.

Ghrelin inhibits GnRH [18] and LH secretions [36] in the pre-pubertal period. Moreover, the ghrelin and its receptors have been demonstrated in mature Leydig and Sertoli cells of rat and human testis [39]. Tena-Sempere [15,37] showed that ghrelin could reduce circulating steroid hormones in pre-pubertal male rats.

It is thought that a degree of body fat is required for initiation of puberty and maintenance of reproductive function in mammals. In humans, the influence of nutrition and body composition on puberty and reproductive physiology has long been recognized [40]. CDGP subjects are typically underweight for height, either due to decreased energy intake [41] or increased energy expenditure [42]. In our study the BMI of the adolescent boys with CDGP were lower than that of the control group, it could be concluded that adipose tissue is unable to maintain adequate leptin production when a higher leptin secretion is required.

Moreover, previous reports show that ghrelin level is negatively correlated with body mass index [43] and weight loss increases circulating ghrelin levels [44]. This increase in ghrelin level may occur as an adaptive response to correct the abnormal energy status. Ghrelin may function as a signal communicating the nutrition states of the body to the central nervous system, Abou Heif et al. [45] reported that ghrelin could be one of the hormones responsible for the suppression of male reproductive axis in case of negative energy balance. So the inappropriately low leptin and high ghrelin secretions could contribute to the relevant state.

Multiple regression analysis revealed that leptin was negatively correlated with ghrelin. Leptin dose-dependently inhibits ghrelin transcription in vitro [46] and decreases ghrelin release from isolated rat stomach [47]. Komori et al. [48] provided a novel molecular link between leptin and ghrelin signaling through the leptin-induced negative regulatory element-binding protein which is an important regulator of GHS-R expression in the hypothalamus. So leptin and ghrelin, two hormones of opposing metabolic effects, could be considered as permissive signals possibly synergistically in the regulatory mechanisms required for reproductive and sexual fitness.

Ghrelin is the first identified hormone that increases feeding when administered peripherally. Ghrelin administration stimulates secretion of growth hormone, increases food intake, and produces weight gain [49,50]. It remains to be determined whether the administration of ghrelin, ghrelin analogues, or small molecule agonists will be useful to treat conditions such as CDGP.

## Conclusions

From the previous discussion it seems that the adipose-derived hormone leptin and gastrointestinal-derived hormone ghrelin communicate information about metabolic status and body weight to the hypothalamus to initiate puberty, so ghrelin and leptin represent metabolic gate for puberty. Elevated serum ghrelin and decreased leptin concentrations and their associations with reproductive hormones may explain the sexual immaturity in adolescents with CDGP.

## List of abbreviations

CDGP: Constitutional delay of growth and puberty; LH: Luteinizing hormone; LHRH: Luteinizing hormone releasing hormone; FSH: Follicle stimulating hormone; GH: Growth hormone; GHS-R: Growth hormone secretagogue receptor; GHRH: Growth hormone releasing hormone.

## Competing interests

The authors declare that they have no competing interests.

## Authors' contributions

MME drafted the manuscript, conceived the study, and participated in its design and coordination. IAA carried out the laboratory studies, AKE helped to draft the manuscript. All authors read and approved the final manuscript.
